# Identifying the Effects of Age and Speed on Whole-Body Gait Symmetry by Using a Single Wearable Sensor

**DOI:** 10.3390/s22135001

**Published:** 2022-07-02

**Authors:** Antonino Casabona, Maria Stella Valle, Giulia Rita Agata Mangano, Matteo Cioni

**Affiliations:** 1 Laboratory of Neuro-Biomechanics, Department of Biomedical and Biotechnological Sciences, University of Catania, 95123 Catania, Italy; m.valle@unict.it (M.S.V.); giuliarita.mangano@gmail.com (G.R.A.M.); mcioni@unict.it (M.C.); 2 Section of Physiology, Department of Biomedical and Biotechnological Sciences, University of Catania, 95123 Catania, Italy

**Keywords:** aging, wearable sensor, symmetry index, speed, anterior-posterior acceleration, spatiotemporal parameters, locomotion

## Abstract

Studies on gait symmetry in healthy population have mainly been focused on small range of age categories, neglecting Teenagers (13–18 years old) and Middle-Aged persons (51–60 years old). Moreover, age-related effects on gait symmetry were found only when the symmetry evaluation was based on whole-body acceleration than on spatiotemporal parameters of the gait cycle. Here, we provide a more comprehensive analysis of this issue, using a Symmetry Index (SI) based on whole-body acceleration recorded on individuals aged 6 to 84 years old. Participants wore a single inertial sensor placed on the lower back and walked for 10 m at comfortable, slow and fast speeds. The SI was computed using the coefficient of correlation of whole-body acceleration measured at right and left gait cycles. Young Adults (19–35 years old) and Adults (36–50 years old) showed stable SI over the three speed conditions, while Children (6–12 years old), Teenagers (13–18 years old), Middle-Aged persons and Elderly (61–70 and 71–84 years old) exhibited lower SI values when walking at fast speed. Overall, this study confirms that whole-body gait symmetry is lower in Children and in Elderly persons over 60 years of age, showing, for the first time, that asymmetries appear also during teenage period and in Middle-Aged persons (51–60 years old).

## 1. Introduction

The forward progression of the body during human locomotion is accomplished by the alternating cyclic movements of arms, legs and trunk. The level of symmetry between the cyclic movements performed on the right and left body sides can impact on walking quality. For example, gait cycle asymmetries could perturb the stability of the center of mass during body weight transfer from one lower limb to the other and/or during the swing phase [1,2,3].

Bilateral gait symmetry has been found to be a sensitive parameter in identifying the reduced level of walking quality and stability in several pathological gaits, such as in the case of lower limb amputation [4,5,6], in children with cerebral palsy [7,8], in patients with chronic stroke [9,10], in Parkinson’s disease [11] and in osteoarthritis [12]. Only a few studies focused on changes in gait symmetry in healthy individuals, as typical human walking is basically considered symmetrical [13]. However, some physiological factors, such as limb dominance [14] or muscle strength [15], may determine bilateral asymmetries also in healthy population.

The physical changes that occur throughout life may be responsible for physiological gait asymmetries in healthy individuals. This may occur during the development period and in the elderly, when motor control and musculoskeletal efficiency decline. Studies that evaluated gait symmetry at different ages in healthy individuals found conflicting results, with some papers reporting no age-related effects on gait symmetry [11,15,16,17,18], and other studies showing the presence of these effects [19,20,21,22,23,24]. A major difference between the two sets of studies was the procedure to compute gait symmetry: in the first set of studies gait symmetry assessment was based on bilateral differences concerning measurements of local parameters, such as spatiotemporal or joint measures associated with gait cycle, while the second set of studies obtained gait symmetry from changes in the whole-body acceleration during right and left walking steps.

Gait symmetry can be evaluated by several computation methods and using a number of kinematic and kinetic parameters [13]. Computing gait symmetry from local gait cycle parameters implies a limited view of the global picture within which the control and the coordination of human locomotion is implemented. In fact, as the final goal of walking is to move the whole body efficiently from one position to another, a quantitative description of the kinematics and/or the dynamics of whole-body movement during locomotion should be the most appropriate approach to integrate the local elements of the gait cycle and to provide a measure of walking effectiveness.

The suitability of the whole-body gait symmetry to more effectively represent the functional goal of walking could be the reason why the authors adopting this method have found a better discrimination across ages than the researchers who have used local parameters to compute gait symmetry. However, the age-related effects on the whole-body gait symmetry were obtained by comparing a limited age range, such as elderly people above the age of 65 years old compared with a group of young adults (20–30 years old) [19,20,21,22] or restricting the analysis to development period [23,24].

In the current study, we assessed bilateral gait symmetry based on whole-body acceleration, in healthy individuals with an age ranging from 6 to 84 years old. This large extension of age categories, tested in the same protocol, overcomes the limitation in covering a short span of ages by the current literature, and allows to explore more accurately the idea that whole-body gait symmetry can distinguish age-related gait in the healthy population.

In particular, we aimed to provide novel information on some age categories, such as Teenagers (13–18 years old) and Middle-aged persons (51–60 years old), neglected in most gait symmetry studies. The development of gait is typically considered complete at the age of 12–13 years old [16,24,25,26], but the maturation process, including improvements in gait symmetry, could continue during teenage years. On the other hand, the detriment in walking efficiency associated with the elderly has been studied mostly in individuals above 65 years of age, thus there is a lack of information on the Middle-Age [27]. In this case, the reduction in gait symmetry expected above 65 years old, could be already present in individuals with ages ranging from 50 to 60 years old.

To better verify the sensitivity of the whole-body gait symmetry index in capturing gait asymmetries across a wide age range, the participants performed walking trials at different speeds, having a comfortable gait as a reference. As the increase in speed is considered a factor capable of amplifying the functional gait differences across the age groups [28], we expected these differences to widen during fast walking.

## 2. Methods

### 2.1. Ethical Statement

This study was conducted in accordance with the Declaration of Helsinki Ethical Principles and Good Clinical Practices and was approved by the local ethics committee of Catania University Hospital “Policlinico Vittorio Emanuele-San Marco” (n° 209/2019/PO). Participation was voluntary and all participants or the legal guardians of minors, read and signed an informed written consent before starting the study.

### 2.2. Participants

In this cross-sectional study we collected and analyzed data from a population of 137 healthy individuals, divided by age into seven groups: 19 Children (6–12 years old; 9 males), 20 Teenagers (13–18 years old; 10 males), 20 Young Adults (19–35 years old; 10 males), 20 Adults (36–50 years old; 10 males), 20 Middle-Aged persons (51–60 years old; 10 males), 20 Senior persons (61–70 years old; 10 males) and 18 Elderly people (71–84 years old; 9 males). Table 1 reports detailed anthropometric data for each group expressed as average and relative standard deviation. The exclusion criteria were as follows: neurological or orthopedic disturbances, cognitive disorders or any other disorder that could affect balance or mobility. Functional physical assessments were performed by a physiatrist (M.C.).

### 2.3. Experimental Procedure

Prior to the recording, the participants were given standardized instructions and explanations about the experimental protocol so that equipment and rules were the same for everyone. In addition to the instructions, all participants were allowed one or two trial attempts before the data were recorded. All participants performed the gait evaluation with bare feet and with loose and comfortable clothes so that they could move freely. The walking area consisted of a 10 m long by 3 m wide corridor. The path was identified with a line marked with colored adhesive tape at the beginning and at the end of the 10 m. The participants walked along the corridor for three times under each of the three experimental conditions: self-selected comfortable speed (Comfortable), high speed (Fast) and low speed (Slow). We balanced the order of presentation of speed conditions across the participants. This protocol took approximately 20–25 min to complete, with an inter-trial rest interval of 1 min.

### 2.4. Data Collection and Processing

A commercial wearable inertial sensor (BTS G-WALK^®^, BTS Bioengineering, Italy) with dimensions of 70 × 40 × 18 mm, properly applied on the skin at the level of the S1 vertebra, was used during walking. The sensor consisted of a triaxial accelerometer 16 bit/axes (sensor range, ±2 g), a triaxial magnetometer 13 bit (±1200 μT), and a triaxial gyroscope 16 bit/axes (sensor range, ±2000°/s). The signals were sampled with a frequency of 100 Hz and transmitted via Bluetooth to a laptop computer for acquisition and processing, using a dedicated software package (BTS^®^ G- Studio, BTS Bioengineering, Italy). Reliability and concurrent validation of this type of inertial sensor, including the BTS G-WALK^®^ [29] were tested for a large number of walking spatiotemporal parameters, showing that accuracy and robustness were within the limits of agreement [29,30].

For each participant, we collected at least 5 gait cycles per trial, ensuring an adequate quantification of gait performance. The individual mean walking speed for the entire path was computed for each participant, for the three speed conditions.

**Figure 1:**
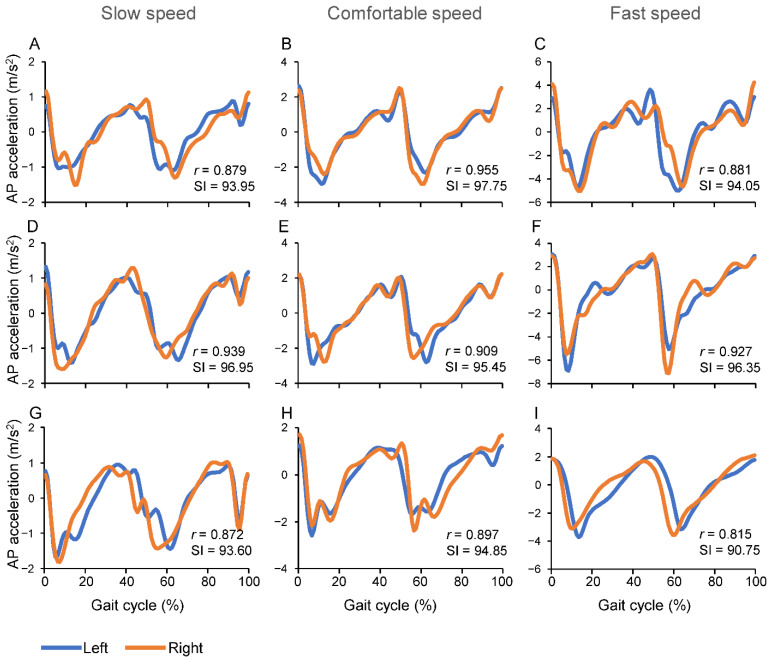
Representative examples of AP accelerations measured in a 9 years old child (**A**–**C**), a young adult 30 years old (**D**–**F**) and an elderly person 81 years old (**G**–**I**). Each plot includes the value of the correlation coefficient (*r*) between left and right gait cycles and the value of the associated gait Symmetry Index (SI).

To estimate the Symmetry Index (SI), we started from the raw acceleration signals captured by the three-axial inertial wearable sensor. After applying a low pass filter (10 Hz cut-off, 2 pass, 4th order Butterworth), the raw acceleration signals were processed as follows:

1. The accelerations due to the sensor movements were separated from the gravity components;
2. The three acceleration axes were rotated from the sensor’s fixed reference frame to the earth reference frame by a trigonometric algorithm [31];
3. From the coordinate system based on the earth reference frame, the anterior-posterior (AP) acceleration was extracted;
4. The individual gait cycles were separated and the single AP accelerations were normalized over the time of each gait cycle;
5. The mean normalized AP accelerations for the left and right cycles were computed (see examples in Figure 1).

From the mean normalized AP signals, the SI was calculated as follows:

1. Pearson’s Correlation Coefficient (*r*) between left and right waveforms of the mean normalized AP acceleration signals was computed;
2. The SI was calculated remapping the values of *r*, ranging from −1 to +1, between 0 and 100 with the following formula: SI = (*r* + 1) × 100/2.

The more the value of SI approaches 100, the more the walking is symmetric (Figure 1).

To evaluate the necessity to normalize the measured data, the SI and the individual speed were first correlated with the weight and the height of the participants. The two anthropometric measures showed significant correlations with the individual speed, but not with the SI. Thus, the individual speed was normalized based on the multiple-regression method as described in Mangano et al. [32]. Briefly, a linear regression model was used to compute the best fit to determine the individual speed, given weight and height. The normalized data were obtained by dividing each original individual speed by the predicted individual speed resulting from the regression model.

### 2.5. Statistical Analysis

The presence of a normal distribution of the data was checked using the Shapiro–Wilk test, while Levene’s test for equality of group variances was used to validate the use of parametric statistics.

The changes in SI and in individual speed were analyzed by performing a two-way Analysis of Variance (ANOVA) with Age group as an independent factor and Speed condition as a factor with repeated measures. The interaction between the factors was also estimated. A one-way ANOVA with repeated measures was used to compare each group across the speed conditions, while a one-way ANOVA for independent measures was performed to compare the groups over each speed condition. When necessary, paired comparisons between groups or speed conditions were performed by a post hoc analysis with Bonferroni correction. For repeated measures, the critical value of F was adjusted applying the Greenhouse-Geisser correction, which produces a more conservative *p*-value.

A linear discriminant analysis was conducted for each speed condition to estimate the contribution of the SI and the individual speed in separating the age groups.

For statistical significance, the level α was established at 0.05.

We used SPSS version 27 (SPSS, Inc., Chicago, IL, USA, IBM, Somers, NY, USA) for statistical analysis, while signal processing was implemented using Matlab version R2022a (Mathworks Inc, Natick, MA, USA).

## 3. Results

### 3.1. Symmetry Index Analysis

Representative examples of changes in AP body acceleration during left and right gait cycles, in a child, a young adult and in an elderly person, are shown in Figure 1. The plots include Pearson’s correlation coefficients (*r*) and the SI values. The main pattern in SI changes was a lower SI value during the Fast speed condition, with respect to the other two speed conditions, for the child and the elderly person, while the young adult showed similar values of SI over the three speed conditions.

**Figure 2:**
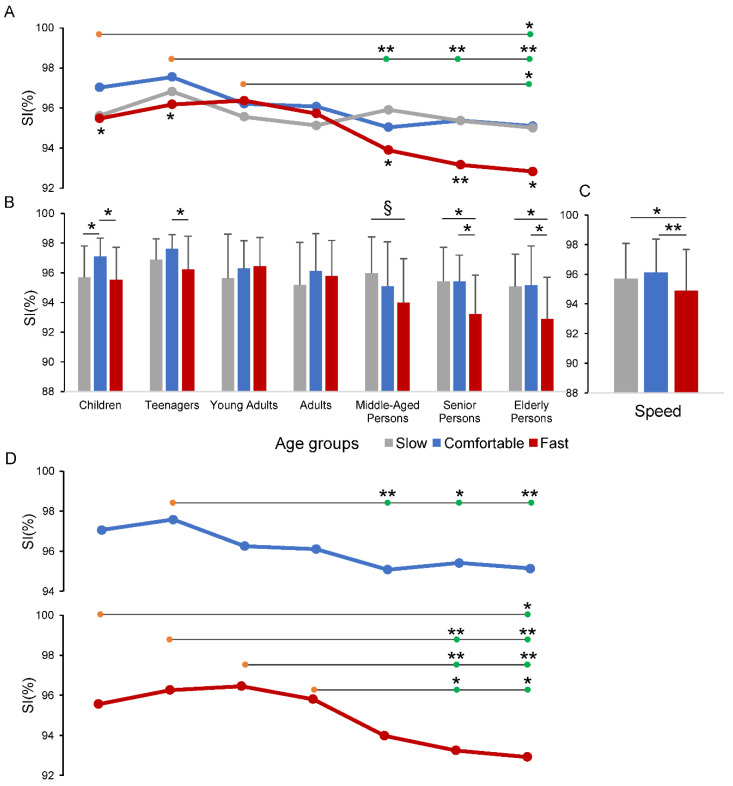
Changes in gait Symmetry Index (SI) across the age groups and speed conditions. In panels (**A**–**C**), the values of SI are showed as lines (**A**) and bars (**B**,**C**) to better capture the changes and the statistical differences across the age groups and speed conditions. Horizontal lines in panels (**A**,**D**) compare one group (orange circles) vs. another group (green circles). In panel (**D**) the Comfortable and Fast speed conditions were extracted from panel (**A**), to report the one-way ANOVA test results for each of the two conditions. The Slow condition is not presented as no statistically significant differences were observed in the age groups. * *p* < 0.05; ** *p* < 0.01; § *p* = 0.068.

The quantitative analysis showed an overall reduction of the SI values in the Children, Teenagers, Middle-Aged, Senior and Elderly persons (Figure 2A,B), particularly for the Fast speed walking, with respect to the other speed conditions. The average group values of the SI ranged from 97.6 to 92.9, with the highest value observed in the Teenager group during the Comfortable speed walk and the lowest value observed in the Elderly persons during the Fast speed condition.

The result of the two-way ANOVA test revealed that changes in SI produced a main effect of the Age group (F_6,130_ = 6.383, *p* < 0.001) and the Speed condition (F_1.9,247.3_ = 12.492, *p* < 0.001) factors, with a significant interaction between the two factors (F_11.4,247.3_ = 2.300, *p* = 0.01). Panel A in Figure 2 and Table 2 summarize the post hoc pairwise comparisons across the age groups associated with the omnibus ANOVA outcome. The horizontal lines in Figure 2A illustrate the pairwise comparisons of one group (orange circles) vs. another group (green circles). Thus, the group of Children and the group of Young Adults showed significant differences in SI with respect to Elderly persons (*p* < 0.05), while the single pair comparisons of Teenagers vs. Middle-Aged persons, Senior persons and Elderly persons showed stronger statistical differences (*p* < 0.01). Numerical data are reported in detail in Table 2.

The post hoc pairwise comparisons for the Speed condition factor (Figure 2C) show that the SI was significantly reduced for the Fast speed condition compared with both Comfortable (*p* < 0.001) and Slow (*p* = 0.011) conditions.

The one-way ANOVA test, with repeated measures, conducted within each group, to compare the speed conditions, is graphically depicted in the panels A and B of Figure 2. These two panels represent the same data illustrated as lines in panel A and as bars in panel B. This representation was necessary to better distinguish the overall statistical differences among speed conditions (indicated by the asterisks in the lower part of panel A) by the pairwise comparison between each of speed conditions (indicated by the asterisks in panel B). The numerical data regarding the omnibus one-way ANOVA for speed conditions and the associated pairwise comparisons are reported in Table 3.

Significant differences in SI values, for the speed conditions occurred for all the groups except for Young Adults and Adults (see asterisks at the lower part of panel A of Figure 2 and the two most left columns of data in Table 3). From these data it can be deduced that the interaction between Age group and Speed condition factors, revealed by the two-way ANOVA, mainly depends on the SI reduction in the Fast speed walking with respect to the other two speed conditions. This occurred, in particular, for the two groups including individuals under the age of 18 years old and for the last three groups with individuals above the age of 50 years old (see the divergence of the red line from the other lines in correspondence with these groups in Figure 2A).

The pairwise comparisons between each of the speed conditions, within each group, are illustrated in Figure 2B and in the three most right columns in Table 3. Most of the significant SI differences there were between the Fast speed and Comfortable speed in Children, Teenagers, Senior and Elderly persons and between Fast speed and Slow speed in Senior and Elderly persons.

The contribution of each isolated Speed condition in discriminating the different age groups was evaluated by a single one-way ANOVA test (Figure 2D). This analysis was conducted on the data shown in Figure 2A, however, for a better identification of the pairwise comparisons, the line plots were duplicated in Figure 2D, except for the Slow speed that exhibited no statistically significant changes.

Significant differences across the age groups were showed during Comfortable (F_6,130_ = 4.105, *p* < 0.001) and Fast (F_6,130_ = 6.894, *p* < 0.001) speed conditions. The horizontal lines in Figure 2D and Table 4 summarize the post hoc pairwise comparisons for the two speed conditions showing statistically significant results. The differences between groups, revealed by the omnibus two-way ANOVA test, appeared to be focused on the SI values measured during the Fast speed condition. In fact, the distribution of the significant differences observed in the Fast speed condition approximately reflects the pattern observed in the post hoc analysis of the omnibus two-way ANOVA test (Figure 2A), with Senior and Elderly persons showing values of the SI lower than in the groups of Children, Teenagers, Young Adults and Adults. On the contrary, in the Comfortable speed condition, only the Teenagers showed significant SI changes with respect to Middle-Aged, Senior and Elderly persons.

### 3.2. Individual Speed Analysis

**Figure 3:**
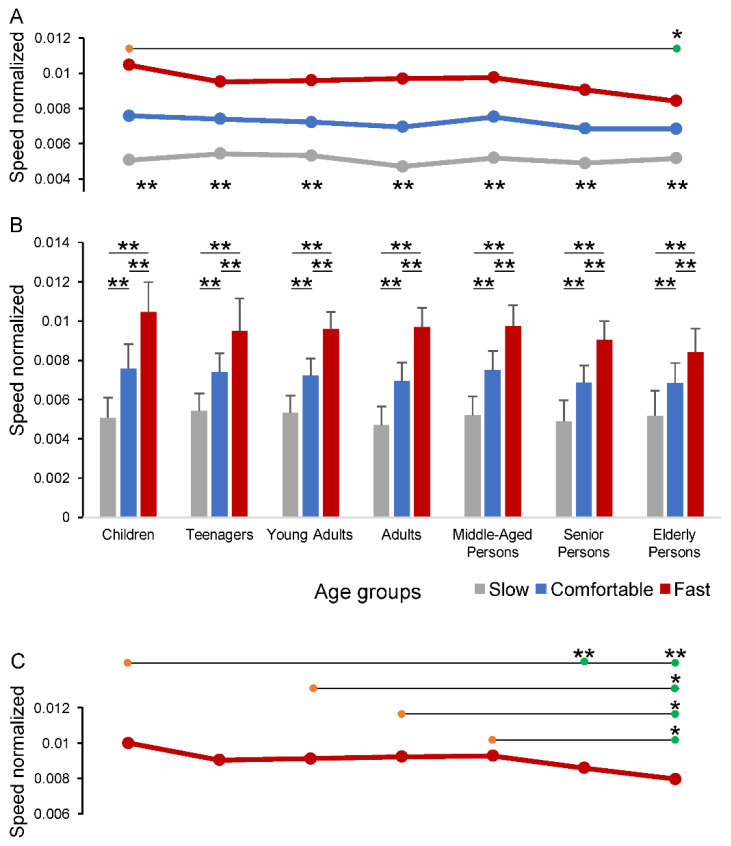
Changes in individual speed across the age groups and speed conditions. In panels (**A**,**B**), the values of individual speeds are showed as lines (**A**) and bars (**B**) to better capture the changes and the statistical differences across the age groups and speed conditions. Horizontal lines in panels (**A**,**C**) compare one group (orange circles) vs. another group (green circles). In panel (**C**) the fast speed condition was extracted from panel (**A**) to report the one-way ANOVA test results for this single condition. The Comfortable and Slow conditions are not presented as no statistically significant differences were observed across the age groups. * *p* < 0.05; ** *p* < 0.01.

The average values of individual speed are shown as un-normalized data in Table 5 and as normalized data in Figure 3. As expected, the values of individual speed changed significantly for the Speed condition factor (F_1.6,205.8_ = 1062.53, *p* < 0.001), with a main effect for the Age group factor (F_6,130_ = 2.733, *p* = 0.016) and an interaction between the two factors (F_9.5,205.8_ = 3.756, *p* < 0.001) as well. The pairwise comparison across the Age groups showed that the overall ANOVA differences were focused between the Children and the Elderly persons (horizontal line in Figure 3A), while each group exhibited significant differences across the speed conditions (asterisks in the lower part of Figure 3A; *p* < 0.001) and between each pair of speed conditions (Figure 3B; *p* < 0.001). The one-way ANOVA test carried out across the groups for separated speed conditions (Figure 3C) revealed that only the Fast speed walking exhibited statistically significant differences, mostly focused between the Elderly persons and each of the other groups, except for Teenagers and Senior persons (horizontal lines in Figure 3C).

### 3.3. Discriminant Analysis

Considering that both the SI and the individual speed showed significant differences across the age groups, we performed a discriminant analysis to quantify the contribution of the two variables in predicting the age changes across the groups. For the Comfortable speed condition, a first discriminant function accounted for 80.2% of group variability, while a second discriminant function accounted for the remaining 19.8%. However, only the first function was statistically significant (Wilks’ Lambda, 0.767; *p* < 0.001) and the structure matrix revealed that factor loading of the SI (*r* = 0.867) was higher than the factor loading of the individual speed (*r* = 0.445). The two discriminant functions were both significant when the discriminant analysis was applied to the Fast speed condition, with the first function having 84.6% of the total variance (Wilks’ Lambda, 0.611; *p* < 0.001) and the second function having 15.4% (Wilks’ Lambda, 0.917; *p* = 0.043). The SI exhibited a higher factor loading than the individual speed for the first function (*r* = 0.745 and *r* = 0.598, respectively), while, for the second function, the factor loading of the individual speed was higher than the factor loading of the SI (*r* = 0.801 and *r* = −0.667, respectively). No significant discriminant functions were detected for the Slow speed condition.

## 4. Discussion

The gait symmetry index based on bilateral whole-body acceleration was lower in Children, Teenagers, Middle-Aged, Senior and Elderly persons than in Young Adults and Adults, during Fast speed condition. Young Adults and Adults maintained a comparable level of whole-body symmetry across the three speed conditions, showing a better ability in gait adaptation with respect to the younger and older participants. It is noteworthy that for the first time, changes in gait symmetry have been identified in Teenagers and in Middle-Aged persons with respect to Young Adults and Adults, suggesting that physiological asymmetries in healthy population can also concern these age categories. Overall, these results indicate that the SI discriminates across the various age groups thanks to the interaction between age and speed factors and provides accurate information on the physiological asymmetries present in healthy individuals.

### 4.1. Local Spatiotemporal Parameters vs. Whole-Body Gait Symmetry Assessments

Local spatiotemporal parameters versus whole-body acceleration symmetry evaluation could explain the different results obtained in the current study with respect to other works where no association was found between gait symmetry and age. In fact, when gait symmetry measures were associated with step length or time, stride length or time, stance or swing time [16,17,18], lower limb joint excursions [11] and muscle strength [15], no significant differences were found across ages, when participants walked at their self-selected speed [11,15,17] or over different speed conditions [16,18]. This dissociation between gait symmetry based on local gait parameters and age may depend on the difficulties of these parameters to capture the complete walking adaptive processes occurring during aging.

It is generally assumed that the adaptations associated with locomotion follow a motor control model where a high level of neuronal elaboration plans the movement trajectory to carry out a purposeful task and a lower neuronal level encodes the rotation of single joint and the muscle forces necessary to execute the plan [33]. A clear example of how this model is implemented is provided by the experimental data reported by Valle et al. [34,35]. These authors, studying locomotion in the cat, identified neurons in the cerebellum whose neuronal discharge was associated with the global kinematics of different walking patterns, suggesting that this activity serves as a basis for the dynamics and the kinematics of individual segments of the lower limbs. In humans, such a process has been extensively studied for reaching and grasping movements where the activity of each muscle and the rotation of single joints, are encoded from a global representation of the trajectory along which the hand moves to reach a position and grasp an object [36,37]. A homologous model can be applied to human locomotion, with trunk and lower limb segments acting to ensure the correct body transfer from one position to another. To accomplish this process, cortical areas, basal ganglia and cerebellum, produce a continuous update of the internal model of body scheme to modulate the motor output from the spinal central pattern generator networks in relation to voluntary intention, and predictive and anticipatory control of locomotion [38]. By this process, the dynamic stability of locomotion is monitored during normal walking and in cases of adaptation to voluntary or contingent requirements [39,40].

Based on the study of Morris et al. [41], gait symmetry showed no association with central cognitive activity in older adults. However, again, the symmetry considered by these authors regarded local characteristics of the gait cycle. To the best of our knowledge, no other studies have focused on a direct relationship between gait symmetry and the activity of central neuronal structures.

The movement of the body’s center of mass is a suitable feature for an internal representation of the whole-body behavior during walking, as the body center of mass motion results from the combination of the complex neurophysiological and peripheral mechanical processes underlying walking [39,42,43]. The position of the body center of mass approximates the position of the second sacral vertebra [44,45], thus, the changes in acceleration recorded by a single inertial sensor applied on the sacral location would parallelly follow the changes in acceleration of the body’s center of mass [31].

Two points reported in the current study suggest that a gait symmetry based on whole-body acceleration may be an indicator for central gait adaptation associated with age. First, most of the differences across age groups occurred during the fast speed walking that can be considered a contingent stress assessment for walking adaptation [28,46]. Thus, the similar SI values reported over the three speed conditions for the Young Adults and Adults indicate a good gait adaptation for these individuals whose gait can be assumed stable. Conversely, the Children, Teenagers, Middle-Aged, Senior and Elderly persons, showed a reduced SI when walking at the fast speed, suggesting difficulties in gait adaptations associated with physical maturation and physical involution, respectively. Second, the discriminant analysis revealed that the SI discriminates across the groups better than gait speed, which is deemed a spatiotemporal parameter sensitive to aging and a useful general characteristic of locomotor control mechanisms [28,46].

### 4.2. Whole-Body Symmetry Assessments and Aging

The idea that the SI based on trunk acceleration may be a candidate to represent age-related changes in walking efficiency, has been supported by several studies [19,20,21,22,23,24]. However, in these studies, limited age segments were examined and trunk acceleration was the basis to make a step-by-step assessment within a single stride [47]. Instead, in the current study, the SI evaluated the bilateral trunk acceleration similarity for each entire gait stride, capturing better the level of coordination of the two alternating limbs during each stride. Our study was designed to evaluate the symmetry index across a large age range in the same experimental protocol and, for the first time, we found that the gait maturation process is still ongoing from children to teenagers and that, although a significant decrease in symmetry occurred over 60 years old, the involution process may start from middle age (51–60 years old).

The typical development period is characterized by continuous physical changes that require constant updating of the body’s internal model to accomplish the associated adaptive processes. In toddlers, at the transition from supported to autonomous walking, gait stability improves with walking practice over a 6-month period [23,48]. Gait maturation continues in the years following the achievement of independent walking, with a complete gait maturation between the ages of 12–13 years old [16,24,25,26]. Regarding the gait symmetry measured in children, Leban et al. [24] evaluating whole-body symmetry based on trunk acceleration, observed that an improved gait symmetry occurred in children aged 12–13 years old with respect to those of 8–9 years old, while Lythgo et al. [16] found that symmetry measures based on several local spatiotemporal parameters were unaffected by age or speed when children (5–13 years old) were compared to young adults (18–27 years old).

In the present study, both Children and Teenagers showed some difficulties in adapting gait, as the SI decreased when walking at the Fast speed condition. This result indicates that the physiological walking development process may not have been completed by 13 years of age, but it may be still in progress over the range 13–18 years old.

As the physiological changes associated with the period of physical maturation accompany improvements of gait symmetry and quality of walking, the natural physical changes in the Elderly persons produce more difficulties in adapting to the Fast speed condition. In the majority of studies, the modifications in walking associated with old age were reported in individuals over 65 years old, without reporting information on middle-aged adults [27]. The data on the SI reported in this paper confirm a lower gait symmetry in individuals above 60 years old with respect to the other age groups. However, a decrease in the SI was also observed in the Middle-Aged persons, (51–60 years old) during the Fast speed walking, although to a lesser extent than in Senior and Elderly persons.

Considering that the participants enrolled in the current study were clinically healthy people, the asymmetries observed can be considered part of the physiological transition toward the end of maturation, in the case of Teenagers, while, in adults over 50 years old, the observed asymmetries would reflect physiological deterioration processes that, in healthy persons, can be compensated to guarantee an acceptable quality of walking.

Overall, the gait symmetry quantified by whole-body bilateral acceleration can be a tool to better discriminate physiological changes in walking across a person’s life span. A lower gait symmetry in Teenagers and Middle-Aged persons, which had not emerged until now, supports the importance of quantifying gait symmetry based on measures that assess the whole movement of the body during walking. Further research will be needed to explore the causes and the implications for which teenagers still yield gait asymmetries and older adults start having challenges in gait symmetry before 60 years old.

## 5. Practical Implications

The knowledge that healthy people show gait asymmetries, even if of minor entity, should be taken in account to distinguish between physiological variations and gait disorders, aiding clinical decision making. The same criteria can be adopted for the selection of control groups in gait analysis laboratories. The results of our study suggest that across the age categories to be considered for a differential analysis, Teenagers and Middle-Aged persons should be included.

As highlighted in the current and other studies [16,21], a critical issue in obtaining consistent differences between healthy and pathological people is to verify the walking performance over three or more speed conditions. In fact, changes in gait speed cause dramatic modifications in kinematics, kinetics and muscle activation patterns [28,46].

It is noteworthy to underline the role that the use of inertial sensors is increasingly playing in quantifying age-related walking parameters, including gait symmetry [13], both in healthy population [19,26,32] and in patients with gait affections [4,5,49]. Our results confirm that the inexpensive and flexible use of a single inertial sensor is also appropriate to quantify gait symmetry during aging, allowing a whole-body symmetry quantification by a simple computational procedure.

Recently, advanced classification procedures, such as machine learning-based algorithms, have relied on data captured by inertial sensors to differentiate clinical scores and to monitor a precision rehabilitation intervention [50] or to differentiate patients from healthy controls for determining the severity of gait disturbances [51]. In this last study, the gait symmetry was one of the relevant measures processed by the machine learning algorithms to determine a reliable severity score of gait disorders.

## 6. Conclusions

Aging can produce gait asymmetry in healthy individuals. The results of this study support the possibility that a bilateral symmetry index, based on whole-body acceleration, may capture the age-related changes when walking adaptations are required to face challenging conditions. In fact, the values of SI are similar across different speed conditions in Young Adults and Adults, which are the age categories representing a reference for the best stability across ages. Conversely, the Children, Teenagers, Middle-Aged, Senior and Elderly persons showed a decrease in SI during the fast speed walking. Particular attention in future studies and in clinical practice is required for the result concerning Teenagers and Middle-Aged individuals, as the physiological variations reported in this study have not been sufficiently emphasized so far.

## Figures and Tables

**Table 1:** Detailed anthropometric data of all participants.

Age Groups	Age Range (years)	Age (years)	Weight (kg)	Height (cm)
Children	6–12	9.3 ±1.9	37.5 ± 12.3	140.8 ± 11.4
Teenagers	13–18	15.5 ±1.7	62.3 ± 10.1	167.5 ± 9.9
Young Adults	19–35	27.6 ± 4.6	67.3 ± 13.1	169 ± 10.6
Adults	36–50	44.8 ± 4.2	68.1 ± 10.7	166.2 ± 8.9
Middle-Aged persons	51–60	56.1 ± 2.8	70 ± 12.67	167.3 ± 9.1
Senior persons	61–70	65.3 ± 3.1	76.5 ± 13.3	164.8 ± 8
Elderly persons	71–84	78.2 ± 3.3	69 ± 11.1	160.3 ± 10.7

**Table 2:** Post Hoc Pairwise Comparisons for the Age group factor associated with the two-way ANOVA test.

Age Groups	Children	Teenagers	Young Adults	Adults	Middle-Aged Persons	Senior Persons
Children						
Teenagers	1					
Young Adults	1	1				
Adults	1	0.341	1			
Middle-Aged persons	0.732	**0.004**	0.613	1		
Senior persons	0.143	**<0.001**	0.11	0.949	1	
Elderly persons	**0.023**	**<0.001**	**0.02**	0.223	1	1

The *p* values were adjusted for multiple comparisons using the Bonferroni correction. Statistically significant values are reported in bold.

**Table 3:** One-way ANOVA test with repeated measures for the speed conditions and post Hoc Pairwise Comparisons between each of the speed conditions.

Age Groups	F	*p*	Comfortable/Fast	Comfortable/Slow	Fast/Slow
Children	5.29	**0.013**	**0.01**	**0.031**	1
Teenagers	3.758	**0.042**	**0.039**	0.227	0.87
Young Adults	0.71	0.469			
Adults	0.99	0.37			
Middle-Aged persons	3.966	**0.034**	0.448	0.4	0.068
Senior persons	6.15	**0.007**	**0.037**	1	**0.034**
Elderly persons	5.525	**0.01**	**0.05**	1	**0.04**

The F values were adjusted applying the Greenhouse-Geisser correction for repeated measures. The *p* values were adjusted for multiple comparisons using the Bonferroni correction. Statistically significant values are reported in bold.

**Table 4:** Post Hoc Pairwise Comparisons, associated with the one-way ANOVA test, between groups for Comfortable and Fast speed conditions.

Comfortable Speed	Children	Teenagers	Young Adults	Adults	Middle-Aged Persons	Senior Persons
Children						
Teenagers	1					
Young Adults	1	0.995				
Adults	1	0.598	1			
Middle-Aged persons	0.08	**0.005**	1	1		
Senior persons	0.332	**0.03**	1	1	1	
Elderly persons	0.131	**0.01**	1	1	1	1
**Fast speed**						
Children						
Teenagers	1					
Young Adults	1	1				
Adults	1	1	1			
Middle-Aged persons	1	0.118	0.058	0.543		
Senior persons	0.115	**0.006**	**0.003**	**0.041**	1	
Elderly persons	**0.047**	**0.002**	**0.001**	**0.016**	1	1

The *p* values were adjusted for multiple comparisons using the Bonferroni correction. Statistically significant values are reported in bold.

**Table 5:** Un-normalized average of individual speed (m/s).

	Slow	Comfortable	Fast
Children	0.72 ± 0.16	1.06 ± 0.16	1.47 ± 0.19
Teenagers	0.91 ± 0.13	1.24 ± 0.15	1.59 ± 0.29
Young Adults	0.90 ± 0.14	1.22 ± 0.13	1.62 ± 1.16
Adults	0.78 ± 0.17	1.15 ± 0.15	1.61 ± 0.16
Middle-Aged persons	0.87 ± 0.16	1.26 ± 0.18	1.63 ± 0.20
Senior persons	0.81 ± 0.16	1.13 ± 0.14	1.49 ± 0.18
Elderly persons	0.83 ± 0.22	1.10 ± 0.16	1.35 ± 0.19

## Data Availability

Not applicable.
